# Ionic Ligand-Enhanced Dual Passivation of Orange CsPb(Br/I)_3_ Quantum Dots for Multicolor Anti-Counterfeiting

**DOI:** 10.3390/molecules31213830

**Published:** 2026-10-23

**Authors:** Fang Lei, Keyujia Zhong, Yujing Xiao, Haohong Chen, Shiqing Dang, Ying Shi

**Affiliations:** 1 State Key Laboratory of Advanced Refractories, Shanghai University, Shanghai 200444, China; 2 State Key Laboratory of High Performance Ceramics, Shanghai Institute of Ceramics, Chinese Academy of Sciences, Shanghai 201899, China

**Keywords:** perovskite quantum dot, CsPb(Br/I)_3_, dual passivation, orange emission, anti-counterfeiting

## Abstract

Dual passivation has recently become a research hotspot for obtaining high-performance perovskite quantum dots (PQDs). However, exploring efficient ligands and their enhancement mechanisms while expanding their anti-counterfeiting applications remains challenging. This work reports a new organic–organic dual passivation combination. The double-passivated PQDs emit stable orange light at 592 nm (under 450 nm excitation). The introduction of a strongly ionic organic compound, sodium 2-bromoethylsulfonate (SBES), improves defect passivation efficiency and increases Fermi levels, thereby effectively separating excitons and suppressing non-radiative exciton recombination. This ultimately more than doubles the photoluminescence quantum yield (PLQY) and simultaneously enhances thermal stability and moisture resistance, with PL intensity retaining 32% and 70% of its initial value after 12 h under thermal and humid conditions, respectively. The characterization of phase, microstructure, photoelectron spectroscopy, and PL dynamics strongly supports these findings. Finally, the fabrication of multicolor films and barcodes combining other PQDs and a long-persistent phosphor validates the potential application of the material system in anti-counterfeiting. This work facilitates the exploration of novel passivation ligands and their enhancement mechanisms for a robust surface engineering approach, and also provides a reference for multimodal luminescent anti-counterfeiting applications based on multi-phosphor combinations.

## 1. Introduction

All-inorganic CsPbX_3_ (X=Cl, Br, I) perovskite quantum dots (PQDs) exhibit exceptional optoelectronic properties, including tunable bandgaps, high photoluminescence quantum yields (PLQY), narrow emission linewidths, strong light absorption, and superior color purity. These unique physical properties render them highly significant for the development of next-generation optoelectronic devices, particularly in the fields of solar energy conversion, lighting, laser, and photoelectric signal detection. Existing studies have demonstrated that the performance metrics of this material system are comparable to or even exceed those of certain traditional semiconductor materials, offering innovative solutions for breakthroughs in advanced optoelectronic technologies [1,2,3]. However, their practical application is hindered by rapid degradation under environmental influences, primarily due to ligand desorption in conventional oleic acid/oleylamine (OA/OAm) systems. This is attributed to the fact that the proton exchange effect of surfactant molecules (OA/OAm) may cause system instability, disrupt the chemical equilibrium of the ligand layer on the surface of CsPbX_3_ nanocrystals, promote dynamic ligand exchange behavior, lead to ligand desorption, increase surface defects, and consequently reduce the quantum yield.

At present, methods for improving the stability of PQDs mainly include ligand engineering, ion doping, core/shell structure design and organic/inorganic hybridization [4,5,6,7,8,9]. Among these, the ligand passivation strategy (ligand engineering) offers significant advantages, as it can effectively passivate surface defects, enhance binding affinity with the substrate, and regulate charge transport.

Typical ligand engineering strategies for CsPbX_3_ (X=Cl, Br, I) QDs reported in recent studies fall into categories such as Lewis acid/base passivation and capping ligand treatment [10,11,12,13]. In one representative work, Zeng and co-workers introduced tetra-n-octylammonium bromide (TOAB) as a Lewis acidic species to coordinate with undercoordinated Pb^2+^ sites and simultaneously compensate halide vacancies, thereby reducing exciton quenching and boosting PLQY to 95% with markedly improved structural stability [14]. Another example is that Sun et al. adopted octylphosphonic acid (OPA) as a Lewis basic ligand to replace conventional oleic acid, also achieving sustained high emission efficiency after multiple passivations [15]. Furthermore, theoretical calculations and experiments have proved that this strategy can maintain the dynamic equilibrium of the ligand layer on the nanocrystalline surface, reduce the surface defect density, significantly improve luminescence efficiency, and provide a new idea for the collaborative optimization of the stability and function of PQD materials [16,17,18,19]. And many researchers have optimized the selection of surface ligands through molecular engineering, giving priority to bidentate or polydentate ligands with strong coordination ability, and introduced steric hindrance groups to enhance adsorption stability [19,20,21,22].

More recently, a dual-ligand synergistic passivation scheme has gained considerable momentum as an advanced frontier in PQD surface ligand chemistry [23,24,25,26]. The core innovation of the dual-ligand strategy lies in the defect-specific division of labor: passivating two chemically distinct types of surface defects on PQDs, namely halide vacancies (e.g., V_Br_) and undercoordinated metal sites (e.g., Pb^2+^), using two complementary ligands with different coordination mechanisms. Therefore, the dual-ligand strategy realizes the simultaneous passivation of both halide vacancies and metal-site defects, which is better than that achieved using single-ligand systems. The resulting charge redistribution from the passivation also generates a built-in electric field that promotes efficient charge separation, which effectively suppresses trap-assisted non-radiative recombination, resulting in significantly enhanced exciton photoluminescence. Furthermore, the direction of Fermi level shift (upward or downward) is highly dependent on the specific chemical nature of the passivating ligands, which also underscores the importance of ligand design in tailoring the electronic properties of PQDs for optimal optoelectronic performance.

As representative ligands to realize the dual-ligand passivation strategy, zwitterionic ligands—such as sulfobetaines, phosphocholines, and γ-amino acids—have attracted interest owing to their bifunctional Lewis acid/base character, which allows simultaneous healing of both anionic and cationic surface traps on PQDs [10,27,28,29]. However, as we know, few studies have exploited halogen-supplying co-ligands for lattice repair [29,30,31,32,33]. Moreover, former applications have mainly focused on traditional fields such as solar cells, lighting and lasers, while few studies have paid attention to anti-counterfeiting technologies such as watermarks, barcodes, and patterns, which are essential nowadays, given the large number of counterfeit and shoddy products that have emerged with the advancement of economic globalization [2,14]. And PQDs offer a promising solution as the main component in invisible monochrome luminescent inks, which can be printed on materials like paper and textiles, and then emit different light under UV excitation to realize multicolor or multimodal fluorescent anti-counterfeiting action [34,35].

In all, further studies on novel zwitterionic ligands and dual passivation mechanisms regarding the luminescence performance of PQDs, as well as their novel optoelectronic applications, are still necessary to improve our understanding in these fields. Herein, based on previous work on double-passivated PQDS using an organic–inorganic combination composed of 3-(N,N-dimethyloctadecylammonium)-propanesulfonate (SB3-18) and SiO_2_ [36], a novel dual passivation design and different optoelectronic properties and potential applications are proposed in this work. Two organic ligands, 3-sulfopropyltetradecyldimethylammonium betaine (STDAB) and sodium 2-bromoethylsulfonate (SBES), were selected to achieve comprehensive defect passivation due to the stronger covalent/ionic bonds than those of oleylamine/oleic acid (OA/OAm), significantly improving the exciton luminescence of CsPb(Br/I)_3_ PQDs together with better humidity and thermal resistance. Furthermore, the potential application in anti-counterfeiting barcodes by integrating the optimized PQDs with other phosphors as multicolor emitters is also demonstrated, which showcases wavelength-dependent encryption capabilities.

## 2. Results and Discussion

**Figure 1:**
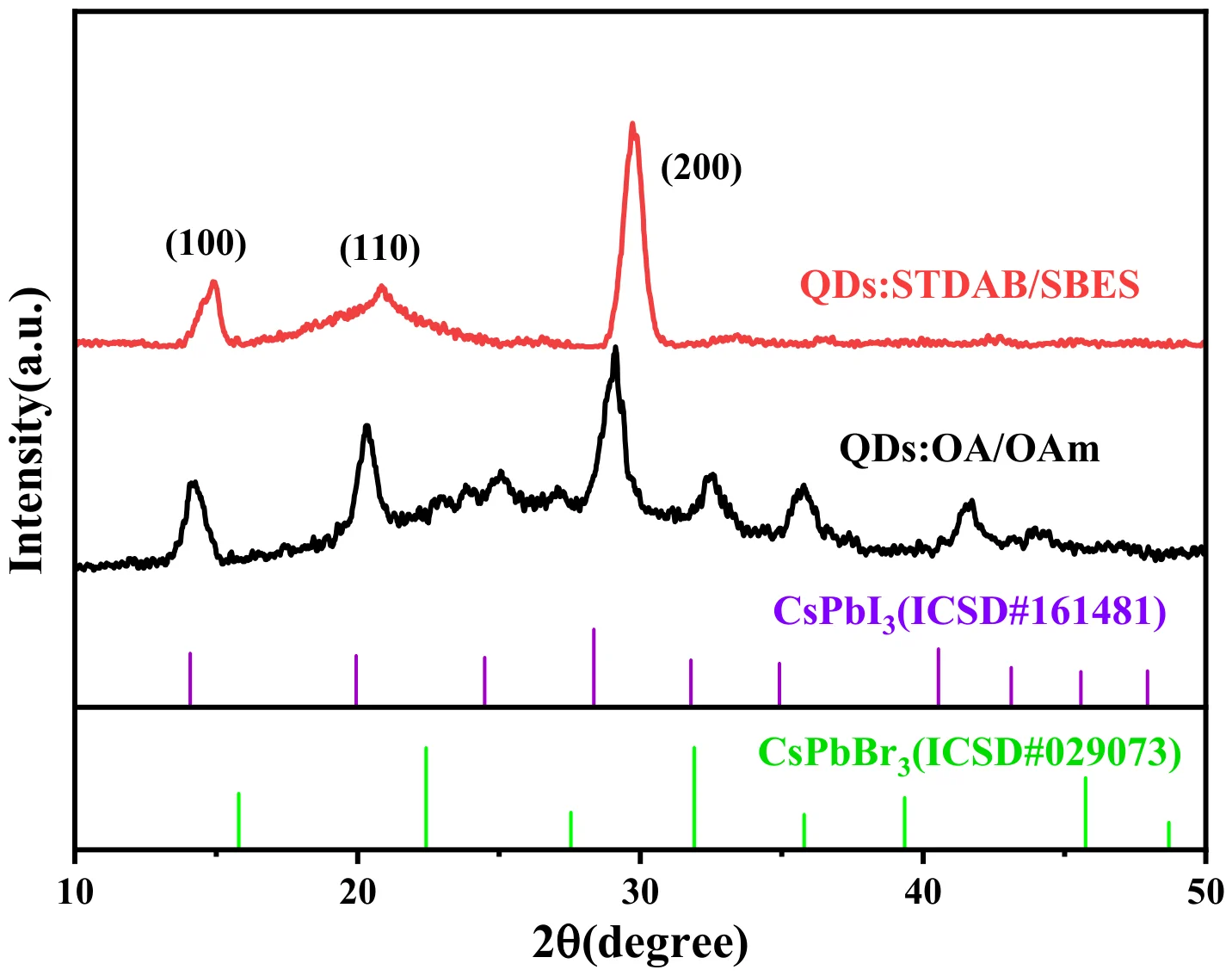
XRD patterns of QDs:OA/OAm and QDs:STDAB/SBES.

**Figure 2:**
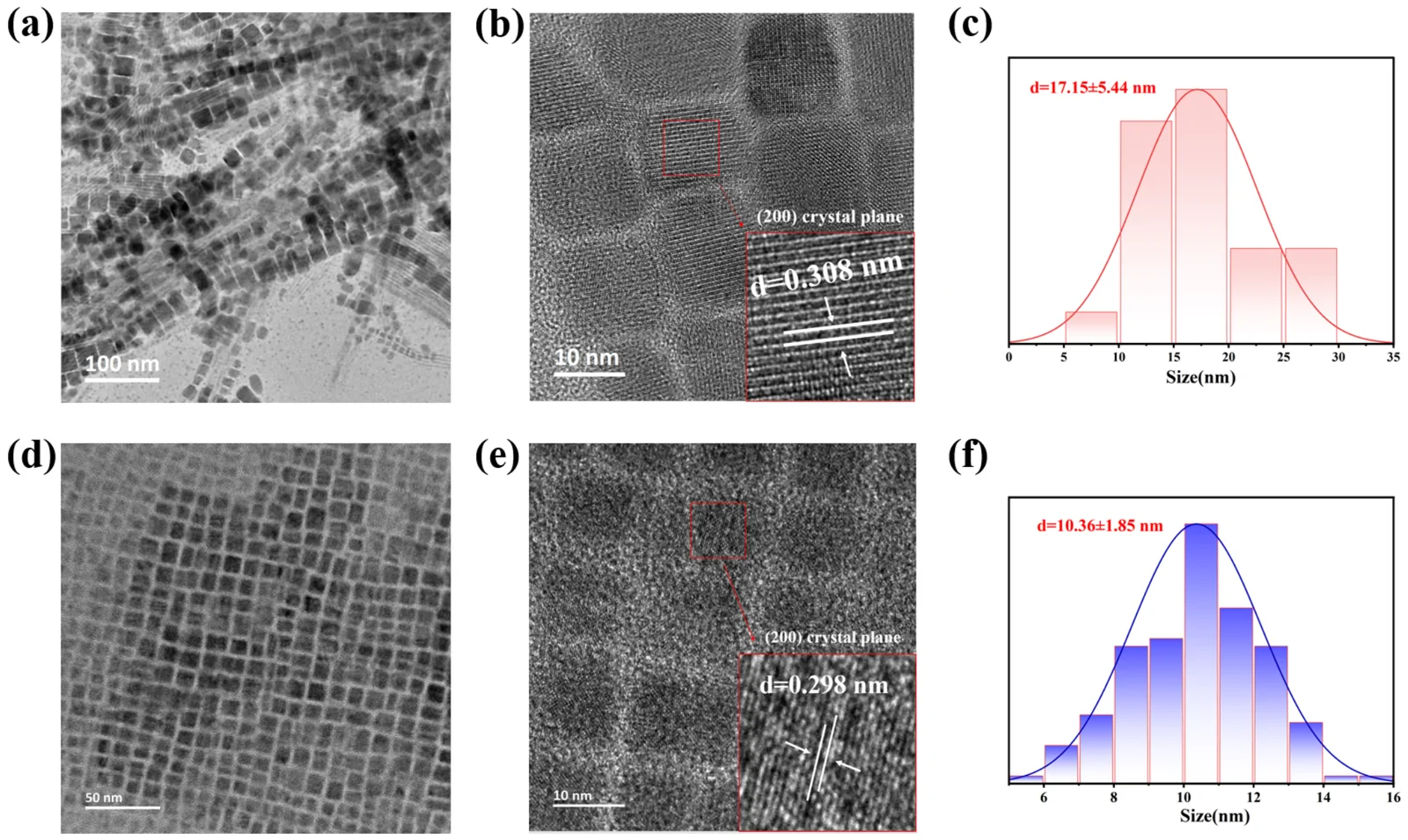
Comparison of TEM/HRTEM morphologies, size distributions, and lattice d-spacings (insets) between QDs:OA/OAm ((**a**–**c**), reprinted with permission from Ref. [36]. Copyright 2026, MDPI) and QDs:STDAB/SBES (**d**–**f**).

**Figure 3:**
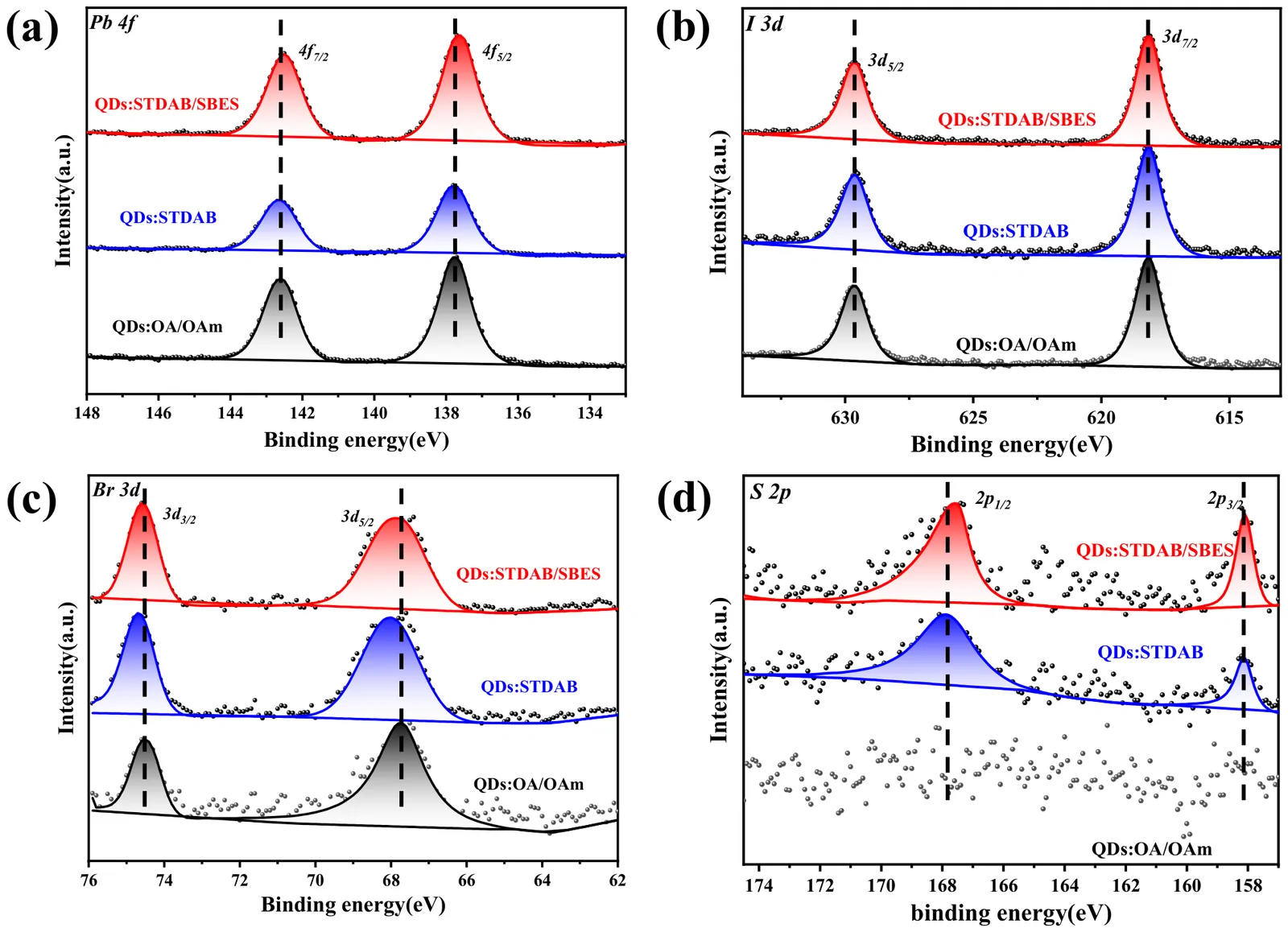
XPS spectra acquired from QDs:OA/OAm, QDs: STDAB, and QDs: STDAB/SBES: (**a**) Pb 4f; (**b**) I 3d; (**c**) Br 3d, and (**d**) S 2p.

**Figure 4:**
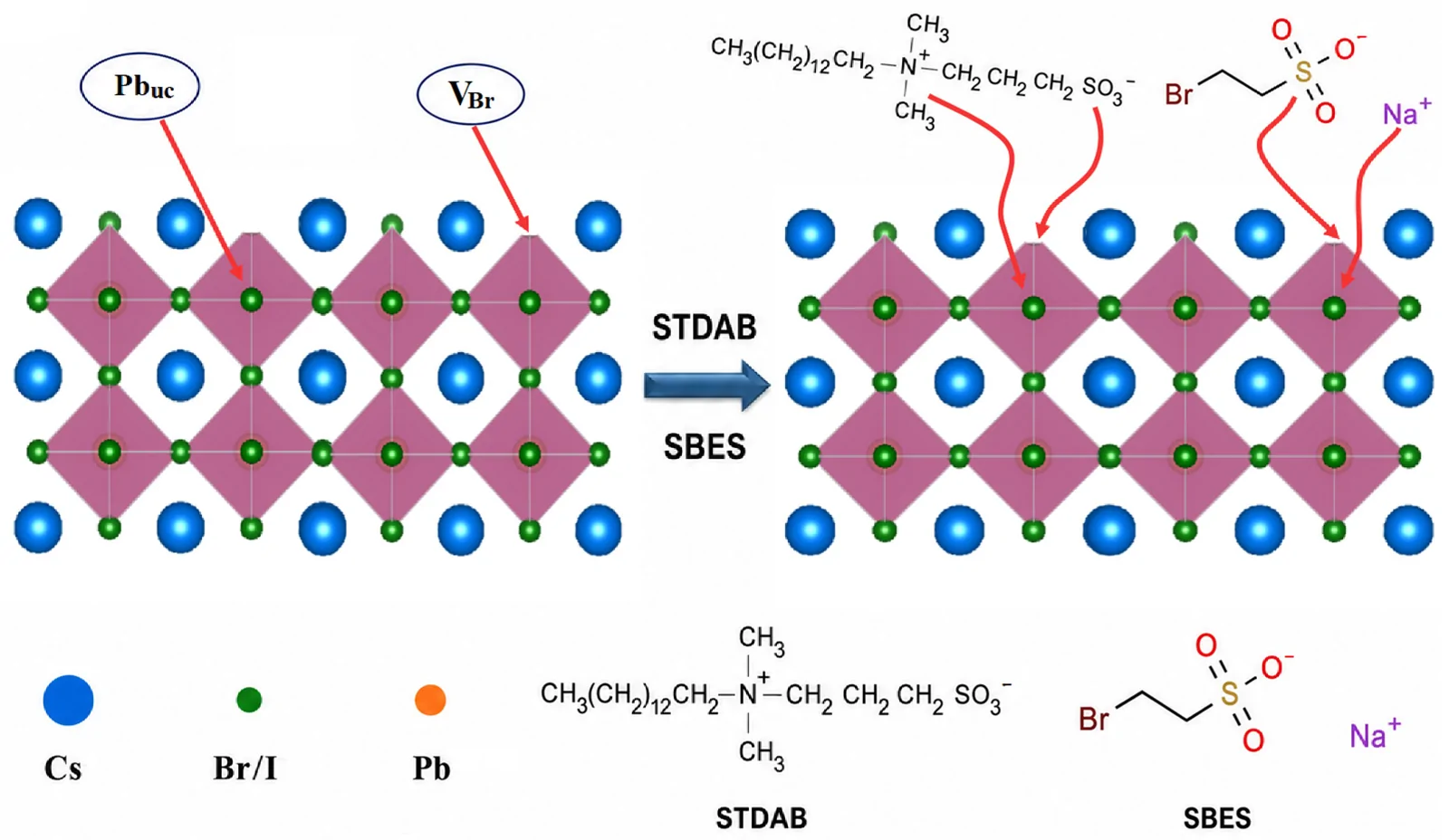
Schematic representation of the proposed passivation mechanisms involving STDAB and SBES. The abbreviations Pb_uc_ and V_Br_ stand for undercoordinated Pb^2+^ ions and bromide vacancies, respectively.

**Figure 5:**
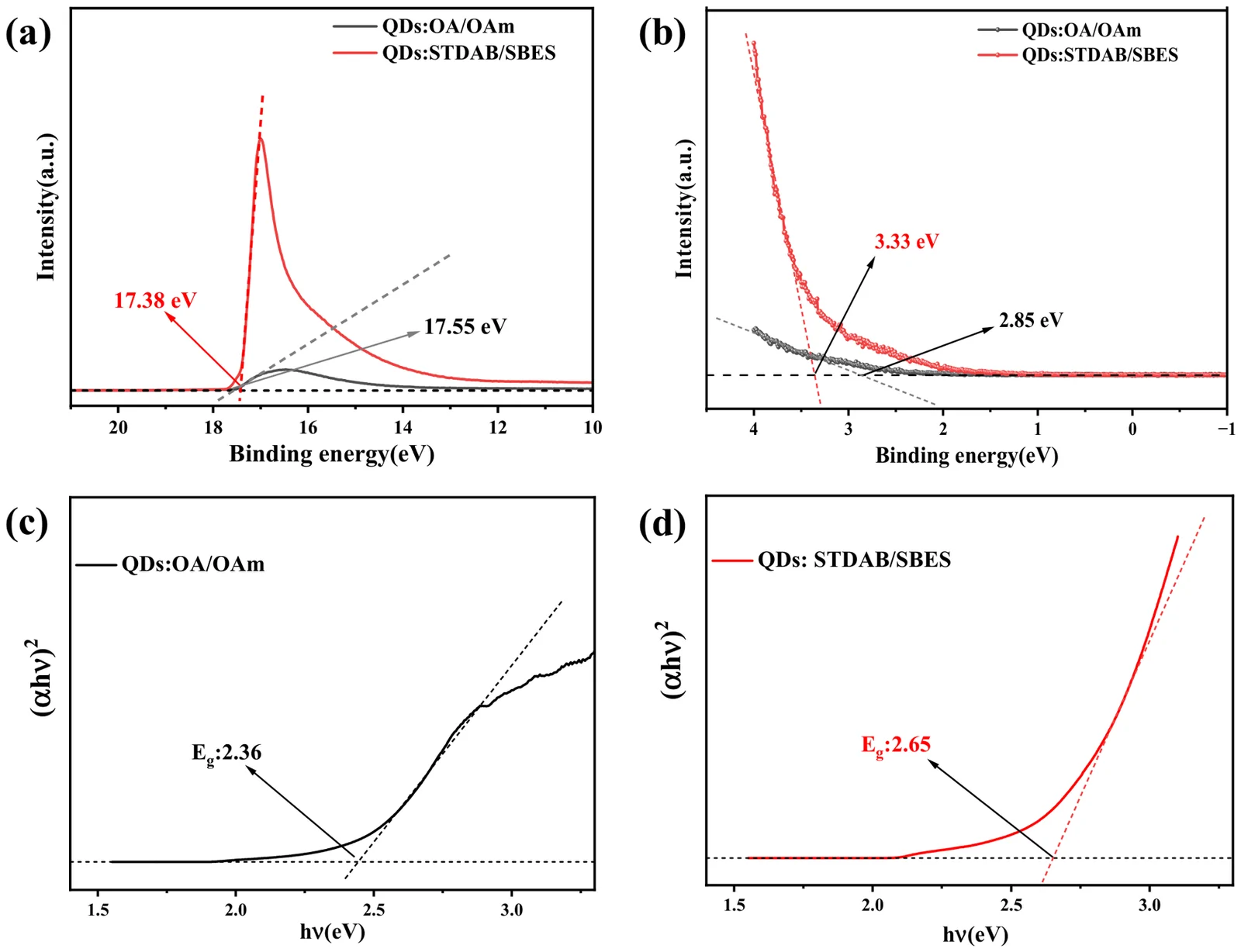
UPS spectra of QDs:OA/OAm and QDs:STDAB/SBES: (**a**) secondary electron cut-off edge, (**b**) near-Fermi-edge (valence) region; and Tauc plots obtained from UV-Vis absorption spectra for bandgap determination of QDs:OA/OAm (**c**) and QDs:STDAB/SBES (**d**), respectively.

**Figure 6:**
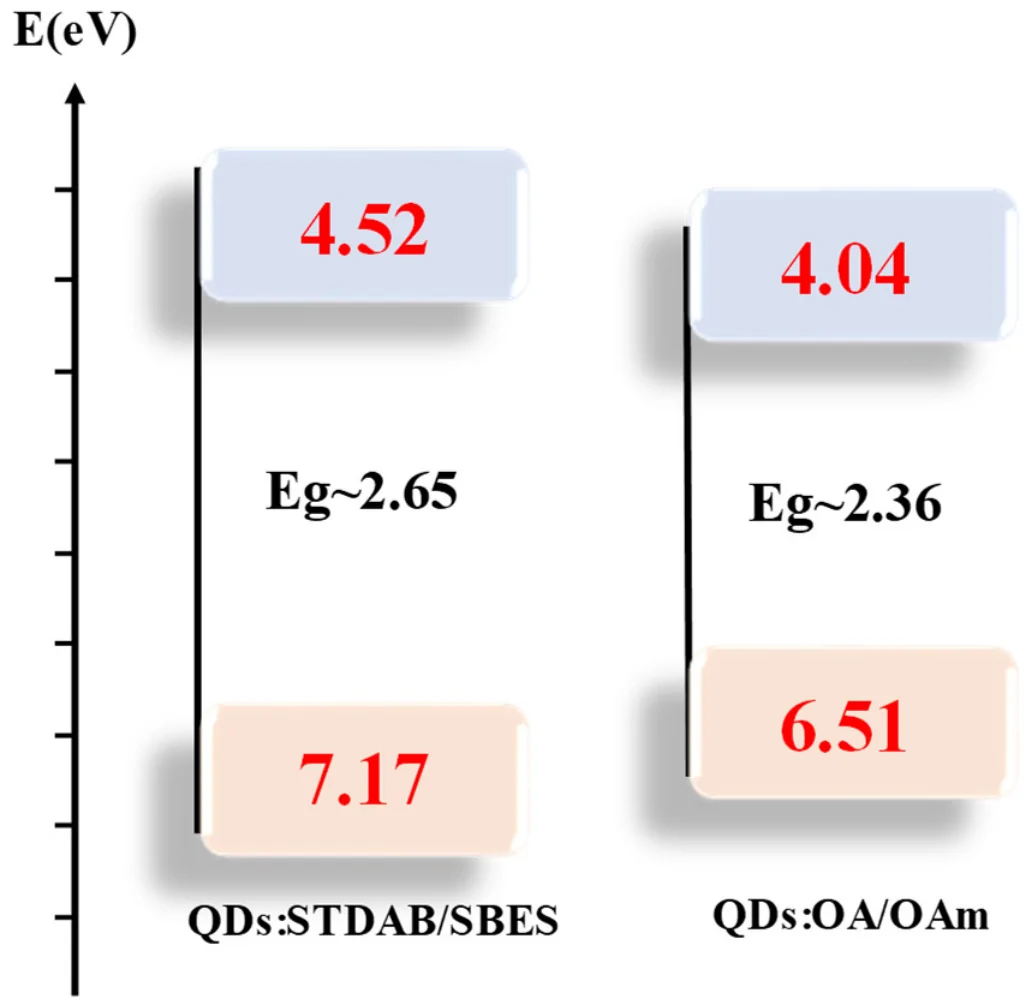
Schematic diagram of the energy levels of QDs:OA/OAm and QDs: STDAB/SBES.

**Figure 7:**
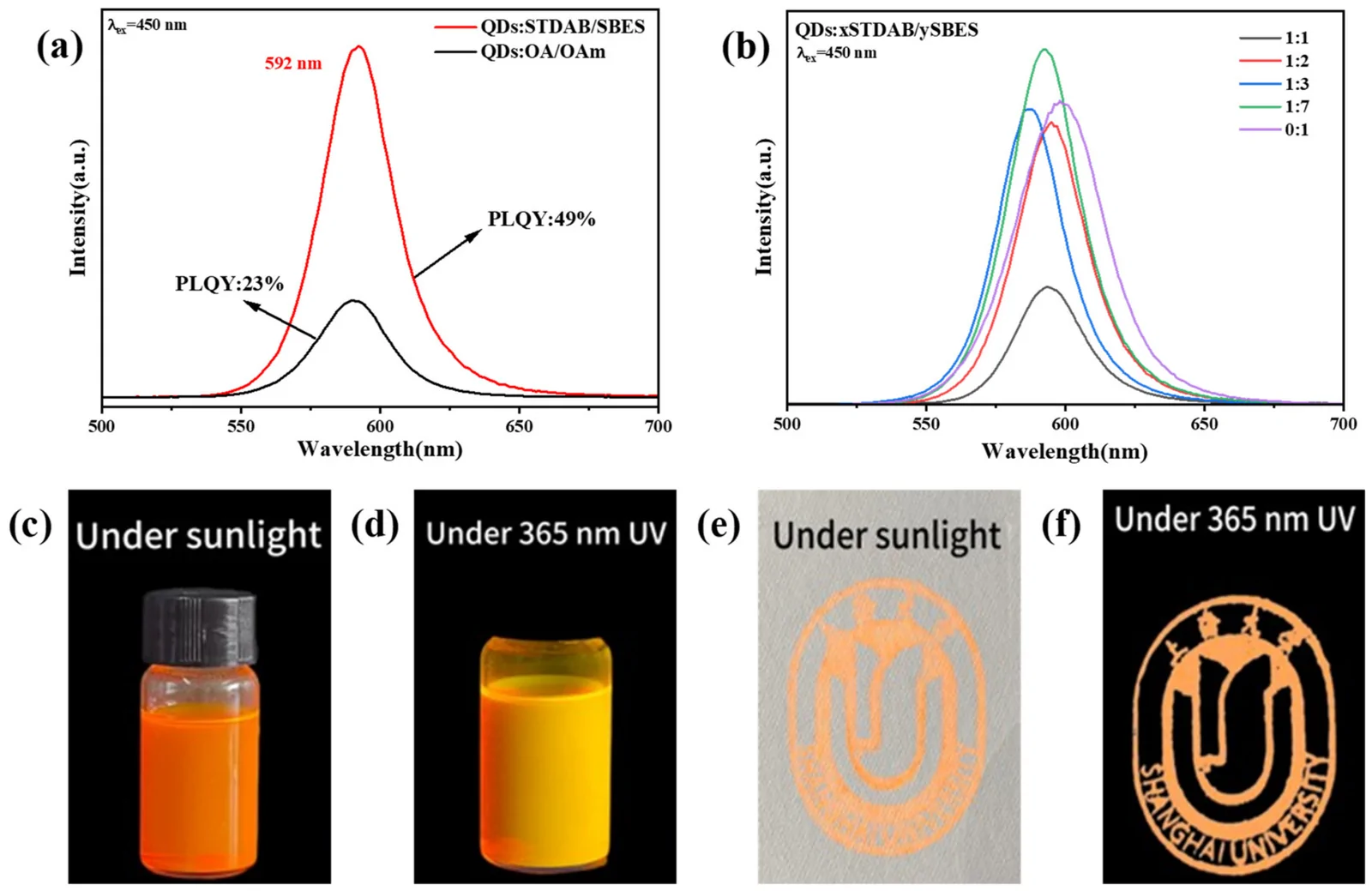
(**a**) PL spectra and corresponding PLQY values of QDs:OA/OAm and QDs:STDAB/SBES: (**b**) comparison of PL spectra of samples with different ratios of STDAB to SBES; (**c**,**d**) photographs of the same QDs:STDAB/SBES dispersion under daylight (**c**) and 365 nm UV (**d**); (**e**,**f**) photographs of the same stamp pattern pained using QDs:STDAB/SBES under daylight (**e**) and 365 nm UV (**f**).

**Figure 8:**
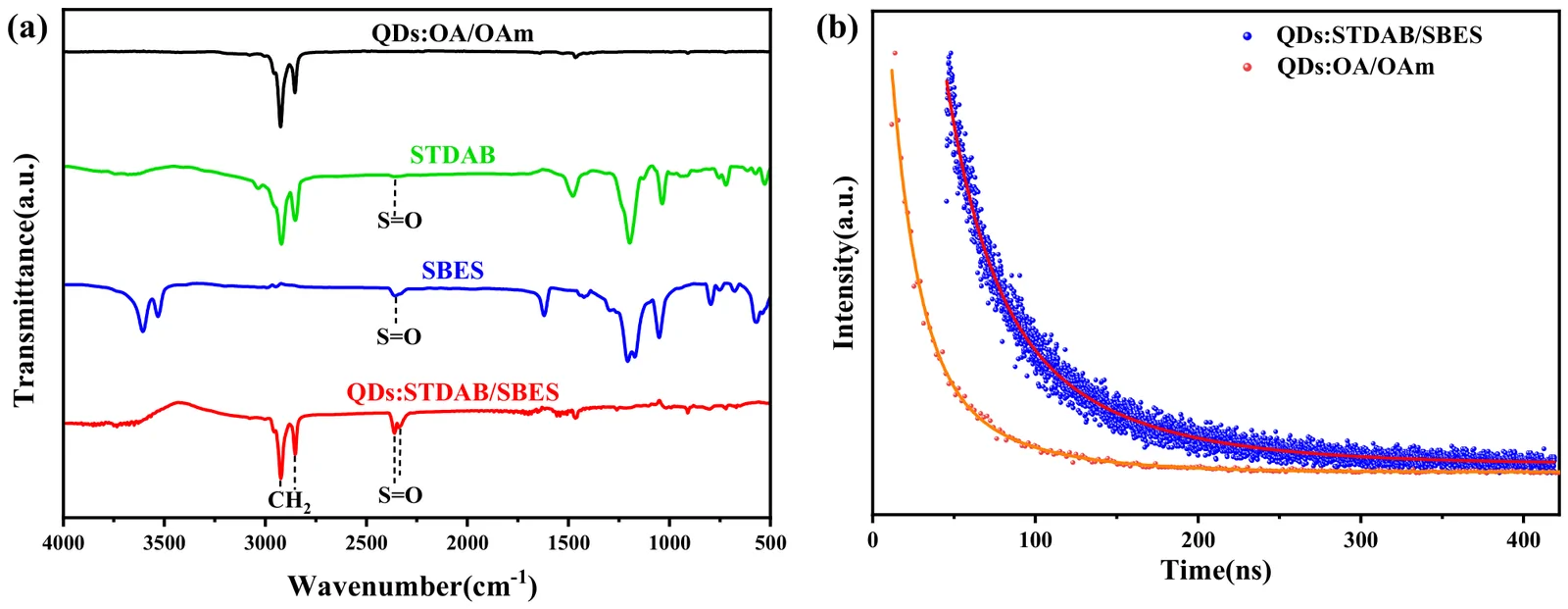
(**a**) FTIR spectra of QDs:OA/OAm, QDs:STDAB/SBES, and the two passivation ligands used (STDAB and SBES). (**b**) PL decay curves of QDs:OA/OAm and QDs:STDAB/SBES.

**Figure 9:**
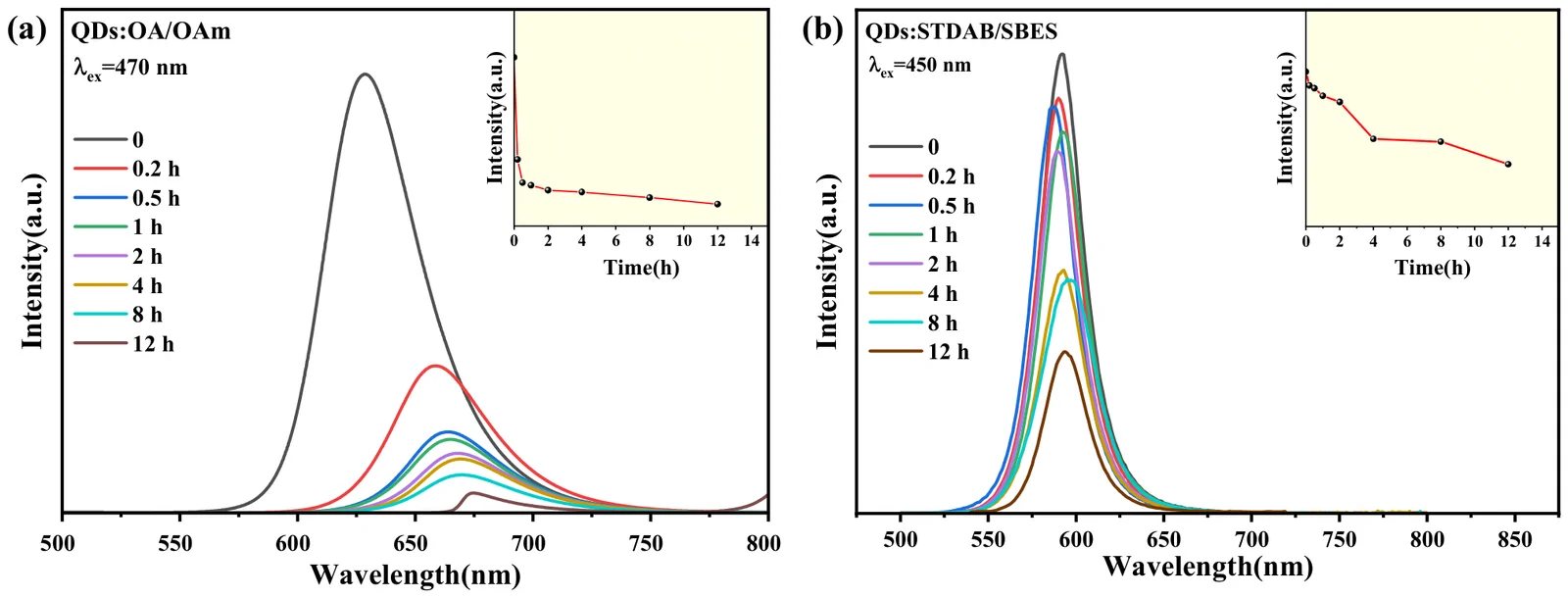
PL spectra of QDs:OA/OAm (**a**) and QDs:STDAB/SBES (**b**) recorded at different time intervals at 80 °C. The insets show the time-dependent evolution of the corresponding PL maximum intensity.

**Figure 10:**
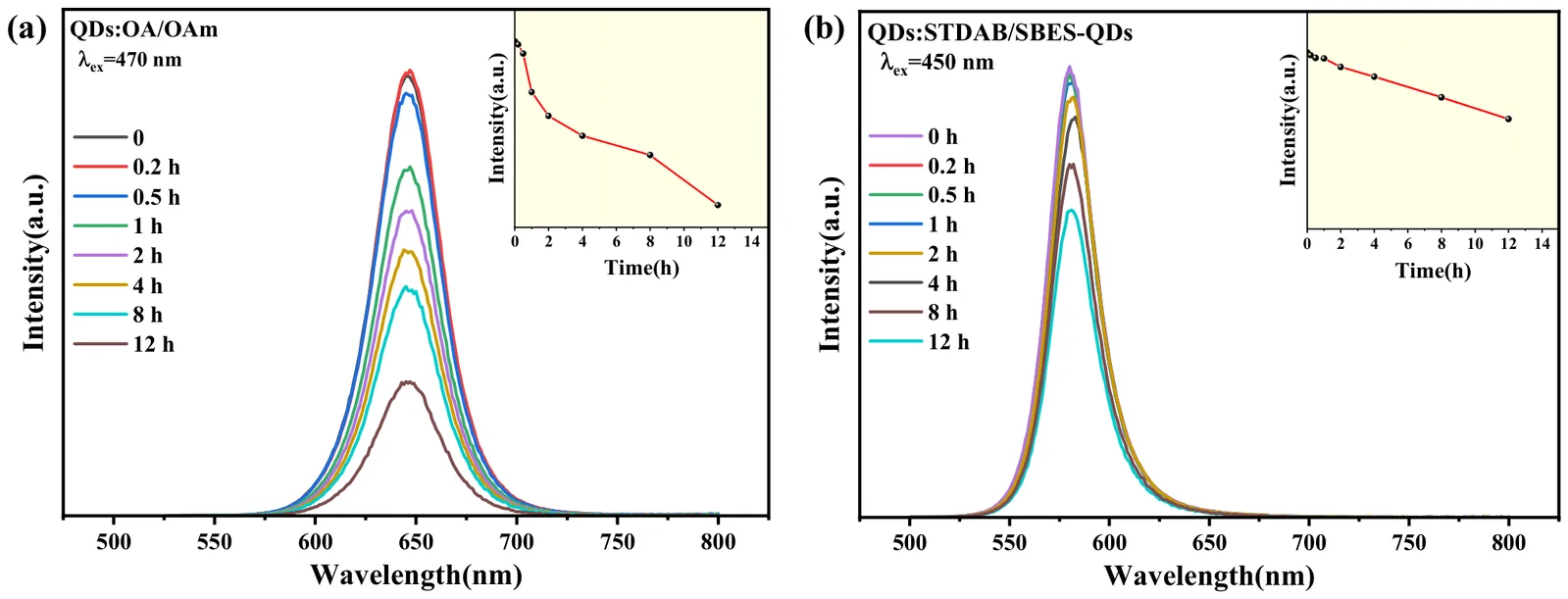
PL spectra of QDs:OA/OAm (**a**) and QDs:STDAB/SBES (**b**) recorded at different time intervals under the same relative humidity (80%) at 25 °C. The insets show the time-dependent variation in the corresponding PL maximum intensity.

Figure 1 presents the XRD patterns of QDs:OA/OAm (without further passivation by STDAB and SBES) and the selected QDs:STDAB/SBES sample with an STDAB:SBES molar ratio of 1:7. The latter was also used as the representative dual-passivated sample for the subsequent characterization and figures including Figure 2, Figure 3, Figure 4, Figure 5, Figure 6, Figure 7, Figure 8, Figure 9 and Figure 10, except for Figure 7a, which includes samples with different ligand molar ratios.

Both samples exhibit pronounced diffraction peaks at approximately 15°, 21°, and 30° (2θ), which are assignable to the (100), (110), and (200) planes of the cubic α-CsPb(Br/I)_3_ phase. In the case of QDs:STDAB/SBES, a broader line profile is observed for the peaks, which indicates smaller particle sizes than those of QDs:OA/OAm. When compared with the standard diffraction pattern of CsPbI_3_ (ICSD#161481), all peaks of the samples are shifted toward higher 2θ values. According to Bragg’s law (2d sinθ = nλ), this angular shift is attributable to the partial substitution of larger I^−^ ions by smaller Br^−^ ions, which reduces the interplanar spacing. This is also supported by the left-shifting of the peaks compared with those of CsPbBr_3_ (ICSD#029073). Consequently, the incorporation of Br^−^ leads to a lattice contraction in the mixed halide QDs.

Notably, the characteristic peak positions of the samples remain nearly identical, indicating that the surface passivation with STDAB/SBES exerts negligible influence on the cubic crystal structures, confirming that the composites have been successfully constructed without altering the perovskite lattice.

To quantitatively evaluate the crystallite dimensions, the average grain sizes were estimated using the Scherrer equation:

$$D=\frac{k\lambda }{Bcos\theta }$$

where *D* denotes the average crystallite size, *k* is a constant (taken as 0.92), *λ* is the X-ray wavelength (1.542 Å), *B* is the full width at half maximum (FWHM) of the diffraction peak, and *θ* is the corresponding Bragg angle. The calculated average grain sizes for QDs:OA/OAm and QDs: STDAB/SBES are 11.38 and 5.08 nm, respectively. This progressive decrease in grain size is fully consistent with the observed trends in peak broadening. Such a variation can be attributed to the additional thermal energy supplied during the post-synthetic passivation processes. The heating steps promote atomic migration and rearrangement at the grain boundaries. Because the short-chain ligands STDAB and SBES possess different steric profiles compared with the long-alkyl-chain OA/OAm ligands, they facilitate greater stability of crystallites of smaller size.

To further investigate the modification effects of sulfonic acid ligands on PQDs, TEM was employed to compare the samples before and after surface treatment. Figure 2a,d show the morphologies of QDs:OA/OAm and QDs:STDAB/SBES, respectively. The QDs:OA/OAm exhibit a certain degree of monodispersity, while the dual-passivated QDs:STDAB/SBES demonstrate significantly improved monodispersity. High-resolution transmission electron microscopy (HRTEM) images (Figure 2b,e) reveal a well-defined cubic morphology and clear lattice fringes, confirming the crystallinity of both samples. The measured (200) lattice spacing is approximately 0.308 nm for QDs:OA/OAm and 0.298 nm for the dual-passivated QDs. The particle sizes determined from TEM images (Figure 2c,f) are 17 nm for QDs:OA/OAm and 10 nm for the dual-passivated QDs. All the varying trends in the results from the TEM/HRTEM images are consistent with those from the XRD pattern. This discrepancy in the particle sizes from the TEM images and the XRD patterns can be attributed to the fact that TEM measures the physical size of individual particles, whereas XRD estimates the average size of coherently diffracting domains (crystallites) within the particles. Therefore, the sizes obtained from XRD patterns are the values of crystal grains, which are normally smaller than the particle sizes determined from TEM/HRTEM images, including the contribution of disordered surface regions. Furthermore, the larger particle sizes observed by TEM/HRTEM also directly indicate the successful deposition of the passivation layer on the quantum dot surface.

Figure 3 presents a comparison of the high-resolution XPS spectra recorded in the Pb 4f, I 3d, Br 3d, and S 2p core-level regions for the QD samples (QDs:OA/OAm, QDs: STDAB/SBES). The relative results of the sample QDs:STDAB were also added for in-depth discussion. As seen in Figure 3a, the Pb 4f peaks at approximately 137.9 eV (4f_7/2_) and 142.7 eV (4f_5/2_), which are characteristic of Pb^2+^ within the [PbX_6_]^4−^ octahedra. For QDs:STDAB/SBES, these Pb 4f peaks undergo a distinct negative shift toward lower binding energies (~137.6 eV and ~142.4 eV, respectively), whereas those of QDs:OA/OAm and QDs:STDAB stay at nearly identical higher energies. Such a shift is indicative of an enhanced electron density around the Pb centers. This observation can be rationalized by the action of SBES as an additional passivating agent: more electron-donating sulfonate groups (-SO_3_^−^) from both ligands coordinate to surface undercoordinated Pb^2+^ ions, increasing the electron density around Pb and enhancing core-hole screening. The effect can be enhanced by employing ligands that form stronger ionic bonds. For example, SBES, which exhibits stronger ionic bonding than STDAB, facilitates the easier dissociation of its Na^+^ ions from the anions in solution, resulting in stronger passivation action of the anions. This also accounts for the negligible shift of the Pb XPS peak when STDAB is employed as the sole passivator, in contrast to the spectra recorded for OA/OAM passivation.

The I 3d peaks (Figure 3b) exhibit no significant binding energy shift across all three samples, confirming that iodine ions are not directly affected by the passivation agents. Conversely, the Br 3d peaks in Figure 3c shift to a higher binding energy after passivation with STDAB and the additionally introduced SBES, indicating a decrease in electron density around Br atoms. The XPS spectral analysis reveals that the surface defects are mainly ascribed to bromide vacancies (V_Br_), while the iodide species are found to be comparatively stable within the PQDs. The Br 3d XPS peaks are shifted to higher binding energies upon treatment with either STDAB or additional SBES, which is ascribable to the electrostatic interactions between surface Br^−^ ions near V_Br_ and the positive ammonium group (-N^+^R_3_) or Na^+^ cations present in these ligands. This reduces electron density around the halides through inductive effects.

Furthermore, the S 2p spectra (Figure 3d) reveal new peaks in the passivated samples (QDs:STDAB and QDs:STDAB/SBES). The S 2p_3/2_ peak at ~168 eV and S 2p_1/2_ peak at ~169.2 eV confirm the presence of sulfur on the surface, originating from the sulfonate groups (-SO_3_^−^) in both STDAB and SBES ligands. The absence of S in the QDs:OA/OAm sample confirms that the S signal arises solely from the passivators. The detection of these peaks demonstrates the successful anchoring of the sulfonate-containing ligands to the QD surface. Compared with the constituent elements of the PQDs (Pb, I, and Br), the S XPS peaks suffer from substantial noise due to the low surface coverage of sulfur-based moieties. This is corroborated by the different extents of shift observed for the two S 2p peaks upon further addition of SBES as the passivating agent, as determined by peak fitting. Accordingly, the current peak fitting serves primarily to verify the existence of sulfur-containing groups at the QD surface, without extending to further quantitative analysis.

Based on the Lewis acid–base reaction theory and the different charged states of the defects, the selective passivation mechanism of specific defects can be reasonably illustrated in Figure 4. The dominant surface defects in the three PQD samples are bromide vacancies and undercoordinated Pb^2+^ ions. These two defect species are coupled based on the requirement of charge neutrality and their formation mechanism. The positive ammonium group (-N^+^R_3_) and Na^+^ can act as Lewis acids to bind to the negatively polarized region around unsaturated Pb^2+^ (arising from neighboring V_Br_+), while the negatively charged groups of the ligands, such as sulfonate groups (-SO_3_^−^), are Lewis bases (electron donors) that engage in coordination with the undercoordinated Pb^2+^ ions. It must be noted that the final electron density around Pb and Br ions largely depends on the iconicity and steric hindrance of the ligands. Unlike the previous work using a longer alkyl group (SB3-18), the decreasing steric hindrance of the ammonium group (-N^+^R_3_) in STDAB can attract more electron cloud directly from the surface of the PQDs to compensate for the charge balance when its sulfonate groups coordinate with Pb^2+^ ions. This reduces the electron density around Br ions and results in a positive shift in the Br 3d XPS peaks. The Na^+^ ions enhance this effect, even causing obvious shifts in the Pb 4f peaks to lower energy. Therefore, the dominant surface defects can be effectively suppressed by the addition of STDAB. Meanwhile, the introduction of SBES also modulates surface electron density, which is also critical to the exciton luminescence properties of PQDs.

The energy level structures of the QDs:OA/OAm and QDs:STDAB/SBES samples were analyzed by UPS. As shown in Figure 5a, the cut-off edge of the secondary electrons of the PQDs is reduced from 17.55 eV to 17.38 eV after double passivation with STDAB and SBES. The work function (*Φ*), defined as the energy difference between the vacuum level and the Fermi level (E_f_), is calculated as:

*Φ* = *hν* − KE_cut-off_

where *h* is Planck’s constant, *ν* is the photon frequency, and KE_cut-off_ is the maximum kinetic energy of the electron. The energy *hν* of the He I light source is 21.22 eV, and KE_cut-off_ corresponds to the energy of the cut-off edge of the secondary electron. Given the same vacuum energy level as zero, the Fermi level can be represented by the work function value.

The work function (*Φ*) of QDs:OA/OAm is calculated to be 3.67 eV. After dual passivation, the work function of QDs:STDAB/SBES increases to 3.84 eV. This increase in the work function indicates that the Fermi level (E_f_) of QDs:STDAB/SBES has shifted downward relative to the vacuum level (i.e., away from the vacuum level) by 0.21 eV. The larger Fermi level can be attributed to two main factors: one is that the surface undercoordinated Pb^2+^ ions are strongly coordinated with the sulfonate groups (-SO_3_^−^) in STDAB and SBES; the other is that the positively charged ammonium groups from STDAB and the Na^+^ ions from SBES also modulate the surface charge distribution. Analogous to the cooperative mechanism demonstrated by Xiong et al. [37], the passivation process leads to a higher Fermi level, resulting in a stronger tendency for holes to migrate toward a lower Fermi level and a decrease in hole density. This larger Fermi level reduces the hole concentration in the quantum dots, consistent with the XPS observation that the Pb 4f peaks shift to lower binding energies, reflecting an increased electron density surrounding the Pb^2+^ sites due to the electron-donating nature of the passivating ligands. The resulting charge redistribution generates a built-in electric field that facilitates hole transfer while suppressing electron transfer, thereby promoting efficient charge separation. This charge separation effectively suppresses trap-assisted non-radiative recombination and benefits stronger exciton luminescence [36,38].

Figure 5b shows the UPS valence band regions of QDs:OA/OAm and QDs:STDAB/SBES. By observing the starting edge of the high binding energy side of the spectra and using the linear extrapolation method to the baseline, the position of the valence band top (VBM) relative to the Fermi level (E_f_, the reference point of binding energy as 0 eV) can be determined. It can be directly read from the figure that the VBM of QDs:OA/OAm starts at the binding energy of ~2.85 eV, and after double passivation, the VBM starting point of QDs:STDAB/SBES moves to ~3.33 eV. This indicates that after passivation, the valence band top has shifted (deepened) downward relative to the Fermi level by 0.45 eV. The conduction band is primarily contributed by the 6p orbital of Pb, while the valence band is formed by hybridization of the Pb 6s orbital and the halogen p orbitals [18]. To obtain the absolute position of VBM relative to the vacuum energy level, it is necessary to add this relative binding energy to the work function (*Φ*) obtained from Figure 5a. It can be calculated that the absolute position of VBM of QDs:OA/OAm is 6.52 eV, while that of the passivated sample is 7.17 eV. Comparative analysis shows that the double passivation strategy not only causes a higher Fermi level, but also leads to a significant, approximately 0.66 eV rigid downward shift in the entire valence band. This movement of the energy band strongly suggests that the STDAB/SBES passivation layer introduces a unified dipole moment on the quantum dot surface, thereby fundamentally adjusting its surface electronic structure, which is crucial for optimizing energy level alignment and charge transport behavior in the device.

The Tauc plots obtained from the UV-Vis spectra for QDs:OA/OAm and QDs:STDAB/SBES are shown in Figure 5c and Figure 5d, respectively. The fitting exponent for the (αhν) item is 2, which indicates that the two PQDs are indirect bandgap semiconductors. The resulting bandgaps (E_g_) are determined to be 2.36 eV (QDs:OA/OAm) and 2.65 eV (QDs:STDAB/SBES). These values lie within the typical 1.5–3.0 eV range for lead halide PQDs. The widening of the bandgap is consistent with the reduced QD size due to passivation, as observed in TEM, which enhances the quantum confinement effect. Furthermore, the widening of the energy gap also originates from a passivation-induced decrease in surface defect density. In lead halide PQDs, surface defects create mid-gap energy levels that effectively narrow the bandgap; they also serve as traps for photogenerated carriers, facilitating non-radiative recombination. Upon passivation, the reduced defect density suppresses non-radiative pathways and removes these mid-gap states, thereby enlarging the bandgap [39], improving charge transport, and ultimately enhancing exciton luminescence.

From the obtained values of E_g_ and VBM, the conduction band minimum (CBM) positions are consequently calculated to be 4.04 eV and 4.52 eV below the vacuum level, respectively. Figure 6 illustrates the energy level alignment among the two samples. In summary, SBES incorporation further diminishes surface defects and modulates the surface electron density, shifting Pb^2+^ to a more negative state and more strongly suppressing non-radiative processes; both effects contribute to the enhanced luminescence observed in Figure 7a.

Figure 7a compares the PL spectra of QDs:OA/OAm and the optimally passivated QDs:STDAB/SBES under 450 nm excitation. The dual-passivated sample exhibits a significantly enhanced emission intensity and a blue-shifted emission peak at 592 nm (compared with the original OA/OAm-passivated QDs), resulting in orange luminescence. The blue shift is attributed to the larger bandgap shown in Figure 6 [23]. The PLQY increases significantly from 23% for the traditional OA/OAm system to 49% for the dual-passivated QDs. Based on the above characterization and discussion of the XPS and UPS results, this substantial improvement arises from the effective passivation of both undercoordinated Pb^2+^ and bromide vacancies by the combined ligands. Therefore, the reduction in surface defects suppresses non-radiative recombination, allowing more excitons to participate in radiative recombination.

The effect of dual-ligand passivation is influenced by their relative molar ratio. Figure 7b shows the emission spectra of PQDs obtained with different ligand ratios (STDAB:SBES) under 450 nm blue light excitation. As STDAB is added and its amount increases from 0 to 0.1, the luminescence is enhanced, accompanied by a blue shift in the emission peak, and the strongest luminescence is obtained at a ratio of 0.05:0.35. With a further increase in STDAB, luminescence decreases, the emission peak exhibits a red-shift, and the emission intensity at a ratio of 0.2:0.2 drops to one-third of that at 0.05:0.35. This is consistent with the XPS analysis: the introduction of SBES can strongly passivate Pb^2+^-related defects, causing a pronounced shift in the Pb 4f peaks, whereas STDAB, although having little effect on the Pb 4f peaks, can also significantly passivate Br vacancies and participate in modifying the surface electronic states, thereby affecting the bandgap of the PQDs. Therefore, an appropriate addition of STDAB helps enhance excitonic luminescence, but excessive addition of STDAB reduces the amount of SBES, weakening defect passivation and decreasing emission intensity. In addition, the PL peak wavelength varies from 575 to 625 nm, spanning 50 nm in Figure 7b. Based on the excitonic emission mechanism of PQDs and the passivation-induced changes in particle size and bandgap shown in Figure 2 and Figure 6, this variation in emission wavelength or color can be attributed to the quantum confinement effect (i.e., the change in the PQD bandgaps). Furthermore, the bandgap change reflected by the emission peak shift in Figure 7b does not vary monotonically, but instead first increases and then decreases. Based on the discussion of Figure 5 and Figure 6, this can be preliminarily attributed to a transition of the surface defect states from sufficient to insufficient passivation. Finally, Figure 7c–f present photographs of the PQD solution and the stamped pattern, each under daylight and UV illumination (365 nm). The distinct behavior under different light sources and the bright luminescence demonstrate the potential of this material for display and multimodal anti-counterfeiting applications.

Fourier transform infrared spectroscopy (FTIR) was further employed to investigate the surface ligand composition and functional groups of the perovskite nanocrystals (Figure 8a) and to support the above analysis based on XPS results. Compared with the OA/OAm-passivated QD sample, the double-passivated QD sample (QDs:STDAB/SBES) exhibits distinct absorption peaks at 2923 cm^−1^ and 2852 cm^−1^, corresponding to the asymmetric and symmetric stretching vibrations of methylene (CH_2_) groups, respectively. Crucially, two absorption peaks at 2362 cm^−1^ and 2335 cm^−1^ appear only in the spectra of the double-passivated QD sample and pure SBES, whereas they are absent in those of the OA/OAm-passivated QD sample and pure STDAB [24]. These peaks are attributed to vibrational modes associated with the S=O and S-O bonds characteristic of the sulfonate group (-SO_3_^−^). This observation provides direct spectroscopic evidence that the sulfonate group from SBES is successfully bound to the QD surface in the dual-passivated system.

To probe the luminescence kinetics and carrier recombination dynamics, the time-resolved photoluminescence (TRPL) curves of the two PQD samples were measured and are shown in Figure 8b. Surface defects in PQDs (undercoordinated Pb^2+^ ions and bromide vacancies) can introduce trap states at various energy levels, leading to multiple carrier recombination pathways. The decay curves were well fitted with a biexponential function:

$$I\left(t\right)=A_{1}exp\left(-\frac{t}{\tau_{1}}\right)+A_{2}exp\left(-\frac{t}{\tau_{2}}\right)$$

where *I* is the emission intensity, *A*_i_ (i = 1, 2) are constant parameters to represent the contribution of each component to the total emission intensity, and *τ*_i_ (i = 1, 2) are the corresponding decay times. The average decay time τ_aver_ is calculated according to the following equation:

$$\tau_{aver}=\frac{\left(A_{1}\tau_{1}^{2}+A_{2}\tau_{2}^{2}\right)}{\left(A_{1}\tau_{1}+A_{2}\tau_{2}\right)}$$

The fitting parameters derived from the decay analysis are summarized in Table 1. The intrinsic fluorescence of QDs originates from excitonic radiative transitions. For both samples, the emission decay profiles comprise a fast (*τ*_1_) and a slow (*τ*_2_) component. The short-lived component is attributable to exciton recombination mediated by surface states or electron transfer, whereas the long-lived component corresponds to radiative recombination of electron–hole pairs released from deep confined states.

The average decay times (*τ*_aver_) are 39.48 ns for QDs:OA/OAm and 76.65 ns for QDs:STDAB/SBES. The average lifetime of the double-passivated QD sample is nearly double that of the OA/OAm-passivated QD sample. This prolonged average lifetime is ascribed to a passivation-induced decrease in surface defects, which suppresses non-radiative recombination pathways during exciton relaxation.

Moreover, the passivation layer enhances structural integrity and reduces defect density, thereby improving stability against environmental factors such as heat and moisture. [25]. To further evaluate the stability of the QDs obtained by dual passivation, the two samples were dispersed in n-hexane, and their PL intensities were compared under simulated heating (80 °C) and humid conditions (80% relative humidity). For a typical emission intensity measurement of one sample under one selected environmental factor, one of its solutions was excited with blue light at set time intervals. Four solutions were employed, and the results are shown in Figure 9 and Figure 10, respectively. It should be noted that the term “stability” is used here to describe the deviation from the initial emission intensity recorded at the start of the measurement.

Figure 9 presents a comparison of their thermal stability. As shown in Figure 9a,b, within the initial 2 h, the PL intensity of QDs:OA/OAm decreased rapidly to 30% of its initial value, whereas that of QDs:STDAB/SBES decreased slowly and remained at approximately 80%. After 12 h of continuous heating, the dual-passivated PQDs still retained 32% of their initial PL intensity, much higher than that of QDs:OA/OAm (9%). This stability is consistent with the aforementioned XPS analysis and the morphology revealed by TEM. Through more complete defect suppression and surface coating, dual passivation helps stabilize the surface states during heating, thereby maintaining a relatively high PL intensity.

The same optimization mechanism is also manifested in resistance to humid environments (Figure 10). Under 80% relative humidity at 25 °C, the PL intensity of QDs:OA/OAm decreased rapidly with time, dropping by 23% after 1 h and to only 28% of the initial intensity after 12 h. In contrast, similar to its behavior in the heating test, the PL intensity of QDs:STDAB/SBES also decreased slowly with time, decreasing by only 5% within 4 h and still retaining 70% of the initial intensity after 12 h.

**Figure 11:**
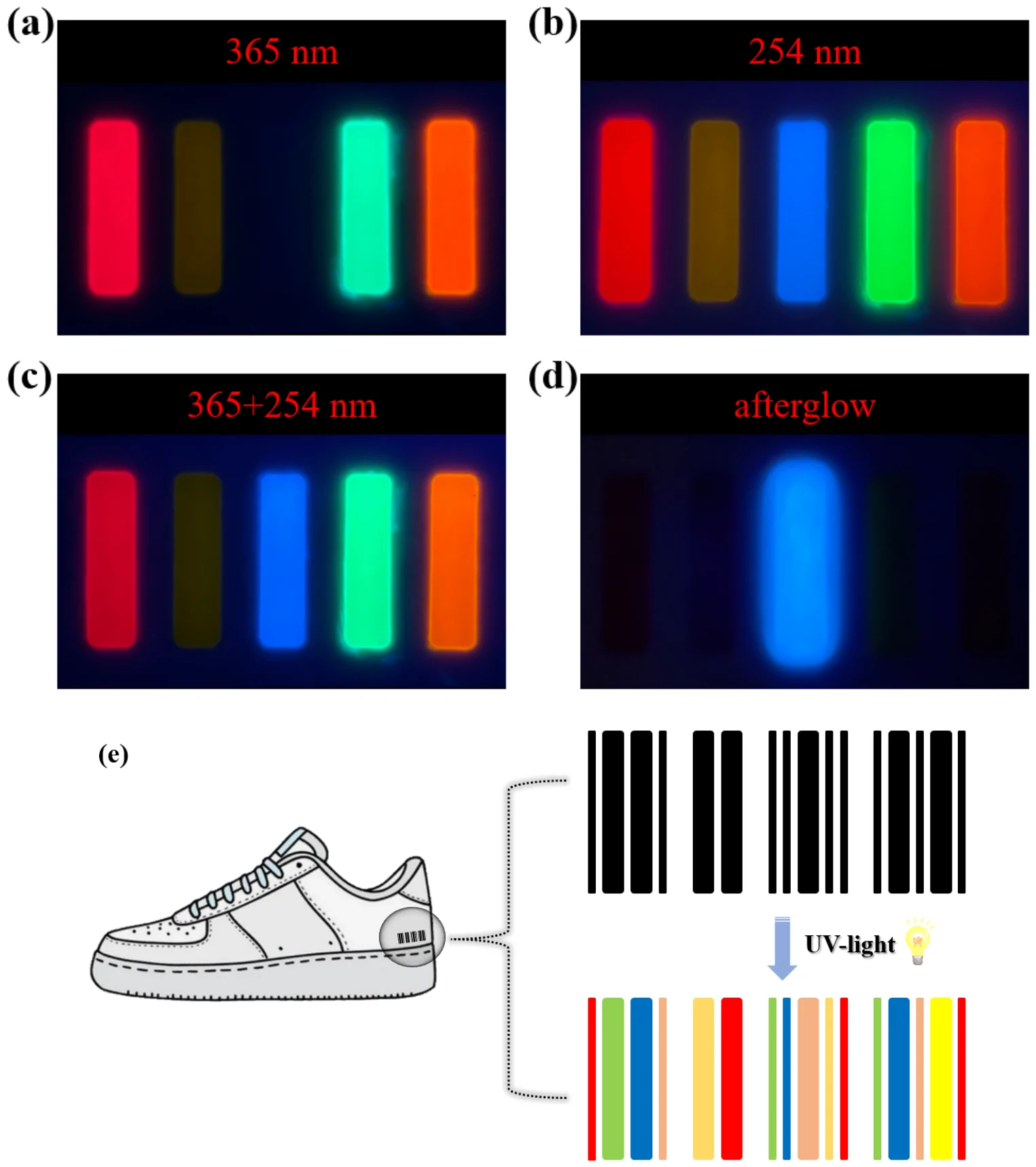
Luminescence images of multicolor anti-counterfeiting design composed of red, yellow, green, and orange PQD colloidal solutions and a blue phosphor under different UV wavelengths: (**a**) 365 nm; (**b**) 254 nm; (**c**) 365 and 254 nm together; (**d**) afterglow of the blue phosphor; (**e**) anti-counterfeiting application as a multicolor barcode on branded sports shoes.

Finally, using QDs:STDAB/SBES as the orange source, Figure 11 demonstrates the luminescent properties of multicolor anti-counterfeiting codes composed of CsPb(Br/I)_3_ colloidal solutions (red, yellow, green, orange) and blue MgGa_2_O_4_:Bi^3+^ phosphors under varying ultraviolet excitation. The other PQDs were obtained with the different ratio of Br:I or Br:Cl and different passivators: (1) SB3-18/SiO_2_ for red CsPb(Br/I)_3_ PQDs (Br:I = 1:2), (2) SB3-18 sole for yellow CsPb(Br/I)_3_ emitter (Br:I = 3:1), and (3) SB3-18 sole for green CsPb(Br/Cl)_3_ PQDs(Br:Cl = 3:1). All five films were prepared using PDMS curing agents. The introduction of the synthesis of the PQDs and the preparation of the films have been reported elsewhere [36]. The blue phosphor was kindly provided by another research group at Shanghai University (see Acknowledgments).

As shown in Figure 11a–d, the composite films exhibit distinct wavelength-dependent responses: under 365 nm UV irradiation (Figure 11a), the system displays four colors with the blue phosphor remaining inactive; at 254 nm excitation (Figure 11b), all five fluorescent components become visible; while simultaneous 365/254 nm irradiation (Figure 11c) produces intermediate emission intensity. Notably, the MgGa_2_O_4_:Bi^3+^ phosphor maintains a detectable afterglow after UV cessation (Figure 11d), providing an additional authentication dimension.

This optical system is designed to potentially address pressing needs in product authentication, particularly for high-value goods like footwear, where counterfeit detection remains challenging (Figure 11e). The implementation involves printing these fluorescent markers on genuine products, enabling verification through simple UV illumination. The authentication protocol leverages three distinct features: the specific color combination under 254 nm excitation serves as primary identification; the wavelength-selective response (absence of blue emission at 365 nm) provides secondary verification; while the persistent afterglow offers tertiary confirmation. Such multi-layered security stems from the unique combination of materials exhibiting (i) non-replicable emission signatures, (ii) excitation-wavelength selectivity, and (iii) time-dependent afterglow behavior. Moreover, all are verifiable using portable UV sources. Therefore, the approach significantly enhances anti-counterfeiting measures by incorporating multiple physical optical characteristics into an easily implementable barcode system.

## 3. Materials and Methods

### 3.1. Materials

Analytical reagents, lead bromide (PbBr_2_), lead iodide (PbI_2_, Aladdin), cesium carbonate (Cs_2_CO_3_), STDAB (CH_3_(CH_2_)_13_N(CH_3_)_2_(CH_2_)_3_SO_3_), OAm, OA, 1-octadecene (ODE), hexane (CH_3_(CH_2_)_4_CH_3_), and ethyl acetate (CH_3_COOCH_2_CH_3_), were ordered from Shanghai Aladdin Biochemical Technology Co., Ltd., Shanghai, China. Analytical SBES (C_2_H_4_BrNaO_3_S) was bought from Shanghai Macklin Biochemical Technology Co., Ltd., Shanghai, China. All raw materials were used as-received without any further purification.

### 3.2. Synthesis of CsPb(Br/I)_3_ PQDs Passivated with STDAB and SBES

The synthesis of STDAB- and SBES-passivated CsPb(Br/I)_3_ PQDs with a Br:I ratio of 1:2 and different passivation ligand contents was conducted via a thermal injection method. The molar ratios of STDAB to SBES used were 0:0, 0:1, 1:1, 1:2, 1:3, and 1:7, respectively, and the sample with a 0:0 ratio corresponded to QDs coordinated solely with OA/OAM (QDs:OA/OAM).

A Cs-oleate (Cs-OA) solution was first prepared by charging a 50 mL three-neck flask with Cs_2_CO_3_ (0.391 g), ODE (19 mL), and OA (1.5 mL). The mixture was degassed under vacuum at 120 °C for 1 h, then heated to 140 °C under N_2_ flow for 30 min until a clear yellow solution formed, which was subsequently stored under refrigeration.

For a typical synthesis of passivated CsPb(Br/I)_3_ PQDs, PbI_2_(0.27 mmol), PbBr_2_ (0.13 mmol), ODE (10 mL), OA (1 mL), OAm (1 mL), and one selected molar ratio of STDAB and SBES were combined in a 50 mL three-neck flask and dried under vacuum at 120 °C for 1 h. The temperature was then raised to 170 °C under N_2_, followed by swift injection of the Cs-OA precursor (1 mL). After 5 s, the reaction was quenched by immersion in an ice-water bath.

The crude PQD dispersion was centrifuged at 11,000 rpm for 10 min, and the supernatant was discarded. The resulting precipitate was washed by adding a mixed solvent of ethyl acetate and hexane (10 mL: 4 mL), followed by centrifugation at 11,000 rpm for another 10 min. The washed precipitate was finally redispersed in 5 mL of hexane and centrifuged at 3000 rpm for 3 min to remove any residual aggregates. The clear supernatant containing the purified QDs was collected for subsequent characterization.

### 3.3. Characterization

Powder X-ray diffraction (XRD) patterns were acquired on a Bruker D8 Advance diffractometer (Bruker AXS GmbH (Bruker Corporation), Karlsruhe, Germany) equipped with Ni-filtered Cu Kα radiation (λ = 1.542 Å). Optical absorption properties were examined over the wavelength range of 300–700 nm using a Shimadzu UV-3600 UV-Vis spectrophotometer (Shimadzu Corporation, Kyoto, Japan). Photoluminescence (PL) spectra were obtained with a Varian Cary Eclipse fluorescence spectrometer (Agilent Technologies, Inc., Santa Clara, CA, USA). X-ray photoelectron spectroscopy (XPS) analyses were carried out on a PHI QUANTERA II system (Physical Electronics (ULVAC-PHI, Inc.), Chanhassen, MN, USA), employing an achromatic Al Kα X-ray source (1486.6 eV) and a double-pass cylindrical mirror analyzer. Fourier transform infrared (FTIR) spectra were recorded on a Tensor-27 spectrometer. Time-resolved PL decay curves were measured using a home-built setup incorporating a 375 nm picosecond pulsed laser. Transmission electron microscopy (TEM) images were taken on a JEM-2100F instrument (JEOL Ltd., Tokyo, Japan). Ultraviolet photoelectron spectroscopy (UPS) measurements were conducted on a ThermoFisher ESCALAB 250XI instrument (Thermo Fisher Scientific Inc., Waltham, MA, USA) with He I_α_ excitation (21.22 eV) as the photon source, a probe spot size of 1–2 mm, and a bias voltage of 10 V applied during the acquisition.

## 4. Conclusions

In summary, we designed a new dual-passivation ligand combination (STDAB and SBES) and successfully obtained orange-emitting PQDs. The more ionic SBES effectively compensates for the limitations of STDAB passivation, enabling simultaneous and efficient passivation of undercoordinated Pb^2+^ ions and bromide vacancies. As a result, the PLQY is more than twice that of the initial OA/OAm-passivated PQDs, and the PQDs also exhibit improved resistance to heat and moisture.

Moreover, the ligand combination enlarges the Fermi level of the system. Varying the molar ratio of the two ligands affects both emission wavelength and intensity, with the emission peak shifting over a range of nearly 50 nm, which provides a new mechanism for tuning the bandgap and luminescence color of PQDs.

Finally, we designed a multimodal anti-counterfeiting strategy that combines multiple emission colors and afterglow colors to produce a complex color-changing anti-counterfeiting scheme that can be implemented using portable UV light sources. A barcode fabricated on branded sports shoes was demonstrated as a specific application. This strategy not only leverages the color advantage of the orange-emitting PQDs obtained in this work but also broadens the approach of combining PQDs with other luminescent materials for applications.

Based on this work, future studies can further strengthen the investigation of how the dual-ligand molar ratio regulates the electronic structure, Fermi level, and bandgap of PQDs, and should also emphasize the influence of the carbon-chain length of the ligands. Overall, this work is expected to greatly promote research into novel high-performance dual-passivated PQDs and their anti-counterfeiting applications.

## Figures and Tables

**Table 1:** Parameters derived from TRPL decay analysis for QDs:OA/OAm and QDs:STDAB/SBES.

Sample	*τ*_aver_ (ns)	*τ*_1_ (ns)	*A*_1_	*τ*_2_ (ns)	*A*_2_
QDs:OA/OAm	39.48	15.61	604.55	54.43	276.93
QDs:STDAB/SBES	76.65	23.96	962.99	76.42	260.44

## Data Availability

The original contributions presented in this study are included in the article. Further inquiries can be directed to the corresponding authors.
